# β-Hemolytic *Streptococcus anginosus* subsp. *anginosus* causes streptolysin S-dependent cytotoxicity to human cell culture lines *in vitro*

**DOI:** 10.1080/20002297.2019.1609839

**Published:** 2019-05-08

**Authors:** Atsushi Tabata, Takuya Yamada, Hiromi Ohtani, Kazuto Ohkura, Toshifumi Tomoyasu, Hideaki Nagamune

**Affiliations:** a Department of Bioengineering, Division of Bioscience and Bioindustry, Graduate School of Technology, Industrial and Social Sciences, Tokushima University Graduate School, Tokushima, Japan; b Department of Biological Science and Technology, Life System, Institute of Technology and Science, Tokushima University Graduate School, Tokushima, Japan; c Graduate School of Pharmaceutical Sciences, Suzuka University of Medical Science, Suzuka, Japan

**Keywords:** Streptococcus anginosus, Anginosus group streptococci, streptolysin S, SLS, β-hemolysis, cytotoxicity

## Abstract

**Background:** Streptococcus anginosus subsp. *anginosus* (SAA) is one of the opportunistic pathogens in humans that inhabits the oral cavity. The type strain of SAA, NCTC10713^T^, showed clear β-hemolysis on blood agar plates, and the sole β-hemolytic factor revealed two streptolysin S (SLS) molecules. SLS is well known as the peptide hemolysin produced from the human pathogen *S. pyogenes* and shows not only hemolytic activity on erythrocytes but also cytotoxic activity in cell culture lines *in vitro* and *in vivo*, such as in a mouse infection model. However, no cytotoxic activity of SLS produced from β-hemolytic SAA (β-SAA) has been reported so far. **Objective and Design:** In this study, the SLS-dependent cytotoxicity of the β-SAA strains including the genetically modified strains was investigated *in vitro*. **Results:** The SLS-producing β-SAA showed cytotoxicity in human cell culture lines under the co-cultivation condition and it was found that this cytotoxicity was caused by the SLS secreted into the extracellular milieu. **Conclusion:** The results from this study suggest that the SLS produced from β-SAA might indicate the cytotoxic potential similar to that of the SLS from *S. pyogenes* and the SLS-producing β-SAA would be recognized as “a wolf in sheep’s clothing” More attention will be paid to the pathogenicity of β-hemolytic Anginosus group streptococci.

The Anginosus group of streptococci (AGS) is a group of streptococcal strains that inhabit the oral cavity in humans. AGS is composed of three species including five sub-species: *Streptococcus anginosus* subsp. *anginosus* (SAA) and subsp. *whileyi, S. constellatus* subsp. *constellatus*, subsp. *pharyngis*, and subsp. *viborgensis*, and *S. intermedius* [1]. More recently, the new subspecies of *S. anginosus* (*S. anginosus* genomosubsp. *vellorensis*) was tentatively advocated from multilocus sequence analysis (MLSA), and the type strain of SAA (NCTC10713^T^ = SK52) was clustered within the *S. anginosus* gssp. *vellorensis* [2]. So, the reconsideration of the type strains of *S. anginosus* subspecies might be done in the near future. AGS is recognized as an opportunistic pathogen in humans, and their pathogenicity has been thought to be low compared to that of the typical human pathogens of Group A streptococcus (*S. pyogenes*, GAS), Group B streptococcus (*S. agalactiae*, GBS), and pneumococcus (*S. pneumoniae*). However, in recent years, studies have shown the pathogenic potential of AGS in humans, such as abscess formation and infectious endocarditis [3–8]. These reports highlight the need for us to re-consider the pathogenic potential of AGS against human.

The pathogenic streptococcal strains produce various virulence factors. One of the representatives is hemolysin. Among the strains belonging to AGS, there are strains that show clear β-hemolysis on blood-agar plate culture. Based on the results from our laboratory and those of other research groups, two kinds of β-hemolytic factors were reported for AGS. One is the intermedilysin, a protein hemolysin belonging to the family of cholesterol-dependent cytolysins (CDCs) secreted by *S. intermedius* [9], and the other is the streptolysin S (SLS), a peptide hemolysin produced by β-hemolytic AGS except *S. intermedius* [10–12]. SLS has been a long-established peptide hemolysin secreted from a human pathogen strain of *S. pyogenes* [13,14], and is reported to be produced as a post-translationally modified product from the precursor encoded by the *sag* operon on the AGS genome. Typical *sag* operons such as *S. pyogenes* are composed of nine genes. However, the *sag* operon in the genome of β-hemolytic SAA (β-SAA) is composed of 10 genes including two SLS-propeptide precursors encoding genes, *sagA1* and *sagA2* [10]. According to our previous report, each of the transcriptional products from *sagA1* and *sagA2* functions as a hemolysin and are secreted into their extracellular milieu during growth [10].

In *S. pyogenes*, the SLS produced is known to be a virulence factor similar to the pore-forming protein hemolysin streptolysin O, and much research was reported on this topic in the middle of the 1960’s and from the late 1970’s to the early 1980’s. However, possibly due to the questionable molecular properties of SLS (e.g., low or no antigenicity and difficulty in purification), the research on SLS temporarily stagnated in the 1990’s. After the year 2000, the situation improved due to the discovery of the genes for the biosynthesis of SLS and their secretion [15]. As a result, many investigations were conducted and SLS was recognized as the virulence factor of *S. pyogenes*. For example, the functions of SLS identified include the inhibition of neutrophil recruitment during the early stages of infection [16,17], translocation across the epithelial barrier [18], contribution of GAS evasion from immune cell killing, local tissue damage, and mortality of mouse [19], promotion of programmed cell death and enhancement of inflammatory signaling in epithelial keratinocytes [20], inhibition of neutrophil recruitment and systemic infection [21], and direct activation of nociceptor neurons and production of pain [22]. In addition, by contribution from the dramatic evolution of next-generation sequencing, the presence of the genes encoding SLS-related peptides in the Gram-positive bacteria besides *S. pyogenes* was also reported. These include the SLS-related peptide hemolysin secreted from *Streptococcus iniae* [23], perfringolysin S secreted from *Clostridium perfringens* [24], and listeriolysin S produced from *Listeria monocytogenes* [25].

For the SLS secreted from the β-hemolytic AGS strains, their function as the hemolytic factor was reported for β-SAA and other β-hemolytic AGS strains [10–12]. However, the cytotoxic potential of the SLS from β-SAA is still inconclusive. The absence of SLS-dependent cytotoxicity of β-SAA against the human cell culture line THP-1 and human granulocytes was reported [26]. However, we had reported previously that the culture supernatant of the β-hemolytic *S. constellatus* subsp. *constellatus* strain W277 showed cytotoxicity to the human hepatocellular carcinoma cell culture line HepG2 [12]. Moreover, in our investigation on the SLS from β-hemolytic AGS strains, we had not ruled out the cytotoxic potential of the SLS secreted from these strains.

Based on the investigation on the SLS produced from β-SAA, we had confirmed the SLS-dependent cytotoxicity of β-SAA in human cell culture lines *in vitro*. In this study, in order to investigate the mechanism of the SLS-dependent cytotoxicity of β-SAA, we used the β-hemolysis and non-hemolysis strains of SAA, NCTC10713^T^ and NCTC11169, respectively. In addition, the genetically modified strains derived from NCTC10713^T^ such as a non-hemolytic NCTC10713^T^ derivative strain lacking both the *sagA1* and *sagA2* genes (Δ*sagA*s), and its *sagA1* gene-complemented strain (p*sagA1*) were constructed for the assay. Using these strains, the evaluation of SLS-dependent cytotoxicity in human cell culture lines was carried out.

## Materials and methods

### Bacterial strains and culture condition

In this study, the type strain of SAA, NCTC10713^T^, showing SLS-dependent β-hemolysis, and a non-hemolytic SAA strain, NCTC11169, were used. In addition to these strains, we tested certain genetically modified strains derived from NCTC10713^T^, an erythromycin (*erm*) resistant strain carrying the inserted *erm* cassette near the upstream region of the intact *sag* operon, a non-hemolytic *erm* resistant strain lacking both the *sagA1* and *sagA2* genes (Δ*sagA*s), and a *sagA1*-gene *trans*-complemented strain of Δ*sagA*s (p*sagA1*). The tested strains were inoculated into BHI broth (Becton Dickinson and Company, Franklin Lakes, NJ) and incubated at 37°C overnight under 5% CO_2_. The human oral squamous cell carcinoma cell line HSC-2 (RCB1945, RIKEN BRC, Ibaraki, Japan) and Eagle’s Minimum Essential Medium (EMEM, FUJIFILM Wako, Osaka, Japan) containing 10% (v/v) of heat-inactivated fetal bovine serum (FBS), 10% (v/v) of BHI, and 50 mM HEPES (pH 7.4) were used for the co-cultivation. The human acute monocytic leukemia cell line THP-1 (RCB1189, RIKEN BRC), and RPMI1640 (FUJIFILM Wako) containing 10% (v/v) of heat-inactivated FBS, 10% (v/v) of BHI, and 50 mM HEPES (pH 7.4) were also used for the co-cultivation.

### Construction of genetically modified strains derived from *S. anginosus* subsp. *anginosus*

The genetically modified strains derived from SAA strain NCTC10713^T^ were generated by the homologous recombination method in the presence of competence-stimulating peptide (CSP) [10]. For the preparation of the genetic fragments for recombination, specific PCR enzymes (PrimeSTAR MAX DNA polymerase, PrimeSTAR HS DNA polymerase, and *ExTaq*; TaKaRa Bio Inc., Shiga, Japan) were selected and used according to the manual. The genetically modified strains were constructed as follows: An *erm* resistant strain was primary generated. To prepare the genetic fragment for homologous recombination, the primer pair of SAA_up_C3-Fw and PstI_SAA_up_C3-Bw (Table 1) was used to amplify the upstream region of the *sag* operon, and the primer pair of BamHI_SAA_dw_C3-Fw and SAA_dw_C3-Bw (Table 1) was used to amplify the upstream region of *sagA1*, using a purified genomic DNA of NCTC10713^T^ as the template. The fragment carrying the *erm* cassette was also amplified using the primer pair of erm_Fw(BamHI) and erm_Bw(PstI) (Table 1), and the purified genomic DNA from the strain possessing the *erm* cassette [10] as template. As per requirement, the amplified fragments were digested using the restriction enzyme(s), *Bam*HI and/or *Pst*I, and purified using the QIAquick Gel Extraction Kit (Qiagen, Hilden, Germany). The purified fragments were ligated using the DNA Ligation Kit (TaKaRa Bio Inc.), and the genetic fragment for transformation was further amplified by PCR using the primer pair of SAA_up_C3-Fw and SAA_dw_C3-Bw (Table 1) with the ligation mixture as template. The transformation of NCTC10713^T^ was conducted under the presence of the amplified fragment and CSP, and selected on the erythromycin containing agar plates [10].

**Table 1.** Primers used in this study.

No.	Name	Sequence (5ʹ-3ʹ)*	Purpose
1	SAA_up_C3-Fw	CTTTTCAAGATCGGAAGAGC	Construction of *erm* resistant strain
2	PstI_SAA_up_C3-Bw	AAACTGCAGAGCAAACAATCCATGAGAGTCG	Construction of *erm* resistant strain
3	BamHI_SAA_dw_C3-Fw	GGGGATCCAGGGATAAATTTCTTACAGG	Construction of *erm* resistant strain
4	SAA_dw_C3-Bw	CCTATTTCACAAATATAACC	Construction of *erm* resistant strain
5	erm_Fw(BamHI)	AATGGATCCCCCGATAGCTTCCGCTATTG	Construction of *erm* resistant strain
6	erm_Bw(PstI)	GTACTGCAGCTAATAATTTATCTACATTCC	Construction of *erm* resistant strain
7	dd1_erm_up_IF Bw	TGAAAAAAATGACATAGTTTGTCCTCCTTATAAAATG	Construction of Δ*sagA*s strain
8	sagB-Fw	ATGAGAATGCTAACGAATTACTAC	Construction of Δ*sagA*s strain
9	SA_sagB_inverse-Bw3	AGTCCTTCAACCTGTCTGGC	Construction of Δ*sagA*s strain
10	Seq_erm cassette-Fw	CTTTCTCTCACTCTGAATGG	Sequencing
11	SA_sagB probe-Bw	GCTTGCGGCAATGCTATGAC	Sequencing
12	sagA1-Bw	TTATTTTGTAGGTGCTACGG	Sequencing
13	In Fusion_sagB-Bw	CAGCTAATTAAGCTTTTAAAACTCCTTATTTCCAAC	Sequencing

*The restriction sites are underlined.

For the generation of the non-hemolytic Δ*sagA*s strain, the fragment for homologous recombination was prepared as follows: each component was amplified by PCR using the primer pair of SAA_up_C3-Fw and dd1_erm_up_IF Bw (Table 1) to amplify the upstream region of the *sag* operon, and the primer pair of sagB-Fw and SA_sagB_inverse-Bw3 (Table 1) was used to amplify the *sagB* using the purified genomic DNA of the *erm* resistant strain as template. To prepare the fragment for transformation, fusion PCR was performed using the primer pair of SAA_up_C3-Fw and SA_sagB_inverse-Bw3 (Table 1). The transformation and the selection were conducted as described above.

To confirm the construction of the genetically modified strains, the genomic DNA of the candidate clone was purified by the method reported previously [27] or using DNAiso Reagent (TaKaRa Bio Inc.). The fragment for Sanger-sequencing was amplified using high-fidelity PCR enzyme (PrimeSTAR series, TaKaRa Bio Inc.), and the primer pair of Seq_erm cassette-Fw and SA_sagB probe-Bw (Table 1). The primers used for sequencing were as follows: Seq_erm cassette-Fw, SA_sagB probe-Bw, and sagA1-Bw (Table 1) for the *erm* resistant strain; and Seq_erm cassette-Fw, In Fusion_sagB-Bw, and SA_sagB_inverse-Bw3 (Table 1) for the Δ*sagA*s strain. The sequencing was outsourced to Eurofins Genomics K.K. (Tokyo, Japan).

### Construction of the *trans*-complemented strain

The *sagA1* gene *trans*-complemented strain was constructed based on the Δ*sagA*s strain using the *Streptococcus-Escherichia coli* shuttle vector pMX2 [28]. Briefly, the fragment from the promoter region of the *sag* operon to the end of the *sagA1* gene was digested from the vector constructed previously [10] using *Eco*RI and *Pst*I, and then inserted into the pMX2 cut with the same restriction enzymes. The competent cell of *E. coli* MC1061 was transformed with the vector and selected. Subsequently, the vector was purified using NucleoSpin Plasmid EasyPure (TaKaRa Bio Inc.). After confirmation of the sequence, the vector constructed for the *sagA1*-gene *trans*-complementation was introduced into the Δ*sagA*s strain in the presence of CSP as described previously [10], and incubated on the horse-blood agar plates containing 4 μg/mL of chloramphenicol, and selected by the recovery of the β-hemolytic character.

### Viability assay of HSC-2 co-cultivated with *S. anginosus* subsp. *anginosus*

HSC-2 was incubated in the standard cell-culture medium for HSC-2 [EMEM containing 10% (v/v) of heat-inactivated FBS and antibiotics (penicillin G and streptomycin)] and inoculated with 1–2 × 10^4^ cells/well/100 μL in a 96-well plate, and then incubated for 1–2 d until the cells in each well reached to almost full-confluence. The pre-cultured SAA strains were diluted to OD_600_ = 0.1, 0.01, and 0.001 with the co-cultivation medium without antibiotics, and 100 μL of each suspension was added to HSC-2, then incubated for 4–5 h at 37°C under 5% CO_2_ atmosphere.

After co-cultivation, the supernatant was removed and the HSC-2 cells were washed three times using EMEM; then the microscopic observation was conducted using the IX71 inverted microscope (Olympus, Tokyo, Japan). In addition, the viability of the co-cultivated cells was evaluated using CCK-8 (Dojindo, Kumamoto, Japan) according to the manual provided by manufacturer. At the end of the co-cultivation, 0.1 N HCl was added to the cells without co-cultivation and incubated for a few minutes to prepare the dead cells to be used as background control. After washing with standard cell-culture medium, CCK-8 was added and incubated; then the absorbance at 450 nm (reference wavelength at 600 nm) was measured by the plate reader Infinite M200 (TECAN, Männedorf, Switzerland).

The SLS-dependent cytotoxicity of HSC-2 under the condition of co-cultivation with the SAA strains was also evaluated using the Transwell (Corning Inc., Corning, NY). Briefly, HSC-2 was inoculated in the bottom at 1.25 × 10^5^ cells/well in 24-well plate and incubated at 37°C under 5% CO_2_ atmosphere for 2 d. A suspension of the tested strain prepared by the co-cultivation medium at OD_600_ = 0.01 was inoculated onto the upper layer of the system and incubated for 18 h. After incubation, the upper layer was discarded and the HSC-2 cells were stained with calcein-AM (Dojindo), propidium iodide (Dojindo), and Hoechst 33342 (Dojindo), and then observed using IN Cell Analyser 6000 (GE healthcare, Chicago, IL).

### SLS-dependent membrane disruption of THP-1

The SLS-dependent membrane disruption of THP-1 was also evaluated. THP-1 was re-suspended in the co-cultivation medium for THP-1 and inoculated at 2 × 10^4^ cells/well/50 μL in the clear-bottom-type 96-well black plate (Greiner, Kremsmunster, Austria). Fifty microliters of the tested strain suspension with OD_600_ = 0.02, prepared by the co-cultivation medium for THP-1, was added to the THP-1 and incubated for 4–5 h at 37°C under 5% CO_2_ atmosphere. The evaluation was conducted using the CellTox^TM^ Green Cytotoxicity Assay (Promega, Madison, WI) as described above.

### Evaluation of the cytotoxicity of culture supernatants from β-hemolytic SAA strains against HSC-2

For evaluation, HSC-2 cells were cultured as described above, and the culture supernatants of the tested strains were obtained from the log-phase culture in the co-cultivation medium. The hemolytic activity of SLS produced in the culture supernatant was confirmed before use for this evaluation. The HSC-2 cells were incubated with the culture supernatant for 1 h or 24 h at 37°C under 5% CO_2_ atmosphere. After incubation, the culture supernatants were discarded and 100 μL of fresh standard cell culture medium was added to each well; then, the viability of the cells were determined using CCK-8 (Dojindo) as mentioned above.

### Time-course investigation of the membrane disruption of culture cells by SLS

The damage to the cytoplasmic membrane of HSC-2 was evaluated using both the kits CellTox^TM^ Green Cytotoxicity Assay (Promega) and CytoTox-ONE^TM^ Homogeneous Membrane Integrity Assay (Promega).

The evaluation using CellTox^TM^ Green Cytotoxicity Assay (Promega) was conducted according to the manual of the kit. The co-cultivation of HSC-2 with the tested strains was carried out according to the method described above except for the use of the clear-bottom-type 96-well black plate (Greiner) for fluorescence measurement instead of the ordinary clear plate. At the end of the co-cultivation, Lysis Solution equipped in the kit was added to prepare the dead-cell control. After the addition of 2 × CellTox Green Dye mix and subsequent incubation, the fluorescence of each sample was measured in a fluorescent plate reader Infinite M200 (TECAN) at the excitation wavelength 490 nm and emission wavelength 525 nm.

Another evaluation was also conducted using the CytoTox-ONE^TM^ Homogeneous Membrane Integrity Assay (Promega) according to the manual of the kit. At the end of the co-cultivation, the dead-cell control was prepared according to the manual. The culture supernatant from each well (three wells per assay condition) was collected and mixed, and then centrifuged (15,400 × *g*, 5 min). Fifty microliters of the supernatant (triplicate per assay condition) was dispensed into 96-well black plate (Corning Inc., Corning, NY). Then, the CytoTox-ONE Reagent was added to each well and the plate was incubated for the time indicated in the manual. After addition of the Stop Solution, the fluorescence of each well was measured in a fluorescent plate reader Infinite M200 (TECAN) at the excitation wavelength 560 nm and emission wavelength 590 nm.

### Statistical analyses

Significance of the difference between two mean values was evaluated by Student’s *t*-test or Welch’s *t*-test after F-test using the software R for Mac OS X (version 3.5.1; https://cran.r-project.org/bin/macosx/). The normality of the data was also evaluated by the Kolmogorov-Smirnov test using the same software described above.

## Results

### Co-cultivation with β-hemolytic *S. anginosus* subsp. *anginosus* causes cytotoxicity in HSC-2 *in vitro*

**Figure 1.**
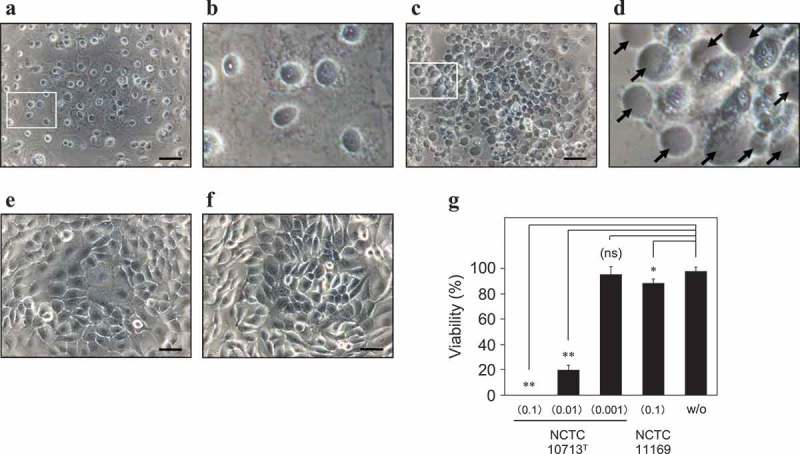
SLS-dependent cytotoxicity of *S. anginosus* subsp. *anginosus* against human oral squamous cell carcinoma cell line HSC-2 under co-cultivation condition. HSC-2 was co-cultivated with higher cell-density (OD_600_ = 0.1) **(a)**, the magnified picture of white square is shown as **(b)**; and moderate cell-density (OD_600_ = 0.01) **(c)**, the magnified picture of white square is shown as **(d)** of β-hemolytic *S. anginosus* subsp. *anginosus* (SAA) strain NCTC10713^T^. HSC-2 was also co-cultivated with higher cell-density (OD_600_ = 0.1) of non-hemolytic SAA strain NCTC11169 **(e)**. Image **(f)** shows the intact HSC-2 without the co-cultivation with bacteria. The morphological observation was carried out using the inverted microscope IX71 (Olympus) equipped with DP72 (Olympus) after removal of the bacterial cells. The scale bar shows 50 μm. The viability of the HSC-2 cells was also evaluated using CCK-8 **(g)**. The results are shown as the percentage of viability with standard deviation (SD) to 100% viable control of HSC-2 without co-cultivation with bacteria (n = 3). The significance of the differences in the results from the cultivation without bacteria (w/o) was evaluated by Student’s *t*-test or Welch’s *t*-test after F-test using the software R (**p < 0.01, *p < 0.05, and “ns“ indicates ”not significant”).

The type strain of SAA, NCTC10713^T^, exhibits β-hemolysis by the production of two SLS molecules with separate amino acid sequences [9]. In order to investigate the SLS-dependent cytotoxicity of β-SAA, the human oral squamous cell carcinoma cell line HSC-2 was co-cultivated with a β-hemolytic strain NCTC10713^T^ or a non-hemolytic strain NCTC11169. The co-cultivation with β-hemolytic NCTC10713^T^ induced obvious morphological changes, such as a flattened morphology probably due to the leakage of the cellular contents (Figure 1(a,b)) and bleb formation probably due to the damage of the cytoplasmic membrane (Figure 1(c,d)) as observed. Contrary to the co-cultivation with β-hemolytic NCTC10713^T^, HSC-2 co-cultivated with NCTC11169 did not show any serious morphological changes and had intact morphology similar to the case without co-cultivation (Figure 1(e,f)). In the viability assay using CCK-8, the HSC-2 co-cultivated with NCTC10713^T^ [OD_600_ = 0.01 or more; multiplicity of infection (MOI) was about 150 or more in this condition] showed a significant (p < 0.01) decrease in viability (Figure 1(g)) compared to the HSC-2 without co-cultivation (designated as “w/o”). In the case of non-hemolytic NCTC11169, the slight but significant (p < 0.05) cytotoxicity was observed by the co-cultivation with a higher concentration of bacteria [OD_600_ = 0.1; MOI was about 890 in this condition]. However, its cytotoxicity against HSC-2 was clearly lower compared to that of the same amount of β-hemolytic NCTC10713^T^.

**Figure 2.**
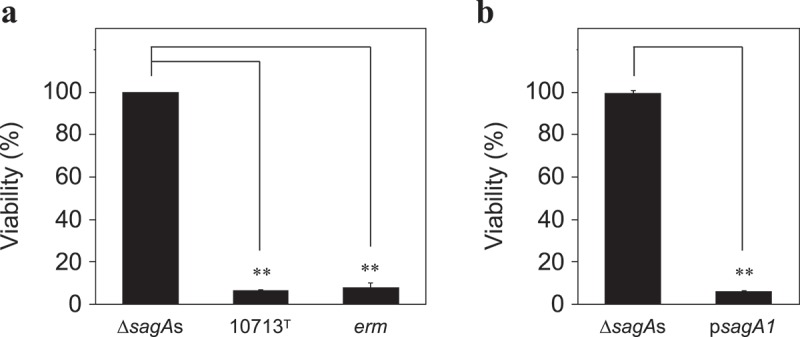
The viability of HSC-2 after co-cultivation with the tested strains. The cytotoxicity of the tested strains (Δ*sagA*s, *sagA1* and *sagA2* double deletion strain of NCTC10713^T^; 10713^T^, β-hemolytic *S. anginosus* subsp. *anginosus* NCTC10713^T^; *erm*, erythromycin-resistant strain of NCTC10713^T^ possessing *erm* cassette on the genome) against HSC-2 was evaluated using CCK-8 **(a)**. The viability of HSC-2 was also evaluated for the *sagA1*-gene *trans*-complemented strain of Δ*sagA*s, designated as p*sagA1***(b)**. The results are shown as the percentage of viability with standard deviation (SD) to 100% viable control of the HSC-2 without the co-cultivation with bacteria (n = 3). The significance of the differences to the result of Δ*sagA*s was evaluated by Student’s *t*-test or Welch’s *t*-test after F-test using the software R (**p < 0.01).

**Figure 3.**
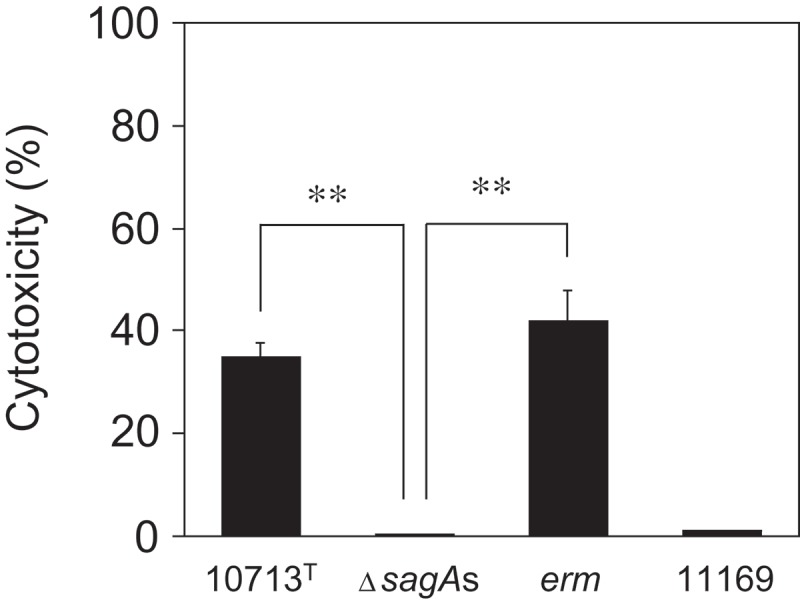
SLS-dependent cytotoxicity of THP-1 after co-cultivation with the tested strain. THP-1 was co-cultivated with *S. anginosus* subsp. *anginosus* strains (10713^T^, β-hemolytic *S. anginosus* subsp. *anginosus* NCTC10713^T^; Δ*sagA*s, *sagA1* and *sagA2* double deletion strain of NCTC10713^T^; *erm*, erythromycin-resistant strain of NCTC10713^T^ possessing *erm* cassette on the genome; 11169, non-hemolytic *S. anginosus* subsp. *anginosus* NCTC11169) for 4 h. Subsequently, the cytotoxicity was evaluated by CellTox Green Cytotoxicity Assay. The 100% live-cell control of THP-1 without the co-cultivation with bacteria as the background was also prepared. The dead-cell control of THP-1 cultured with no bacteria was treated with ‘Lysis solution’ just before the end of the incubation. The results are shown as the percentage of cytotoxicity with standard deviation (SD) to 0% viable control of the THP-1 (n = 3). The significance of differences to the result of the non-hemolytic strain (Δ*sagA*s) was evaluated by Welch’s *t*-test after F-test using the software R (**p < 0.01).

In order to investigate the contribution of SLS produced from β-SAA to the cytotoxicity of HSC-2, the viability of HSC-2 co-cultivated with the Δ*sagA*s strain, the non-hemolytic isogenic mutant strain of NCTC10713^T^, was also investigated. Consequently, the co-cultivation with the Δ*sagA*s strain showed no cytotoxicity, in contrast to both the β-hemolytic strains, NCTC10713^T^, and the *erm* resistant strain (Figure 2(a)). Similar to the HSC-2 co-cultivated with non-hemolytic NCTC11169 (Figure 1(e)), HSC-2 co-cultivated with the Δ*sagA*s strain also did not show any morphological changes with intact morphology of HSC-2 (Figure S1). The SLS-dependent cytotoxicity was further investigated by the *sagA1*-gene *trans*-complemented strain p*sagA1*. From the results, p*sagA1* also showed significant cytotoxicity similarly to the NCTC10713^T^ and *erm* resistant strains (Figure 2(b)). The SLS-dependent cytotoxicity was also investigated for the human acute monocytic leukemia cell line THP-1. In the assay using the CellTox Green Cytotoxicity Assay system (Promega), the SLS-dependent cytotoxicity was also observed in THP-1 similarly to that in the HSC-2 cells (Figure 3). These results indicated that the SLS produced from β-SAA functioned as a factor inducing cytotoxicity.

### Culture supernatant of β-hemolytic *S. anginosus* subsp. *anginosus* is not sufficient to cause acute cytotoxicity in HSC-2

**Figure 4.**
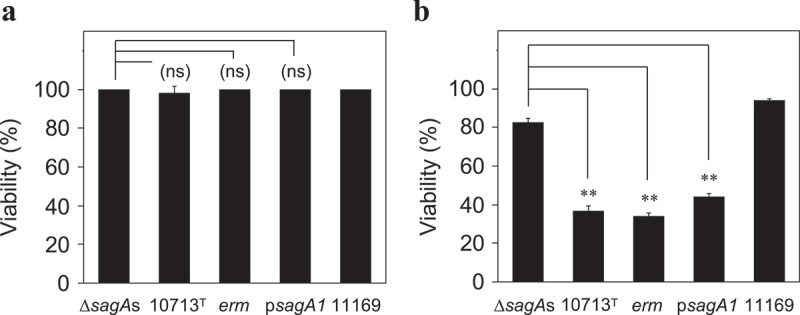
The viability of HSC-2 incubated with the culture supernatant of the tested strains. HSC-2 cells were incubated with the culture supernatant of the tested strains (Δ*sagA*s, *sagA1* and *sagA2* double deletion strain of NCTC10713^T^; 10713^T^, β-hemolytic *S. anginosus* subsp. *anginosus* NCTC10713^T^; *erm*, erythromycin-resistant strain of NCTC10713^T^ possessing *erm* cassette on the genome; p*sagA1, sagA1*-gene *trans*-complemented strain of Δ*sagA*s; 11169, non-hemolytic *S. anginosus* subsp. *anginosus* NCTC11169) as described in “Materials and methods“ and incubated for 1 h **(a)** or 24 h **(b)**, then the viability was evaluated using CCK-8. The results are shown as the percentage of viability with standard deviation (SD) to 100% of the viable control of the HSC-2 with no treatment with bacterial culture supernatant (n = 3). The significance of differences to the result of the non-hemolytic strain (Δ*sagA*s) was evaluated by Student’s *t*-test or Welch’s *t*-test after F-test using the software R (**p < 0.01 and “ns” indicates ”not significant”).

Subsequently, the cytotoxicity of the culture supernatant of β-SAA was investigated. HSC-2 was incubated in the presence of the culture supernatant from a β-hemolytic strain with sufficient hemolytic activity. After 1 h incubation, no significant acute cytotoxicity was observed (Figure 4(a)). However, the prolonged (24 h) incubation induced sub-acute cytotoxicity in HSC-2 (Figure 4(b)).

To evaluate further the sub-acute cytotoxicity of the culture supernatant from β-SAA, the time course of the culture supernatant-mediated sub-acute cytotoxicity was also investigated. Consequently, the decrease of viability of HSC-2 was observed by the 24 h incubation with the culture supernatant of β-SAA with high hemolytic activity, i.e., the culture supernatants prepared from both 4 h and 6 h culture of NCTC10713^T^ (Figure S2a). No decrease in viability of HSC-2 was observed by the incubation with the culture supernatant of non-hemolytic Δ*sagA*s strain at the same incubation time (Figure S2b). Because the growth property of both NCTC10713^T^ and Δ*sagA*s in the co-cultivation medium was almost the same (Figure S2c), the decreased viability of HSC-2 incubated with the culture supernatant of NCTC10713^T^ was thought to be caused by the secreted SLS.

### Cytotoxicity of β-hemolytic *S. anginosus* subsp. *anginosus* was caused by the secreted SLS

**Figure 5.**
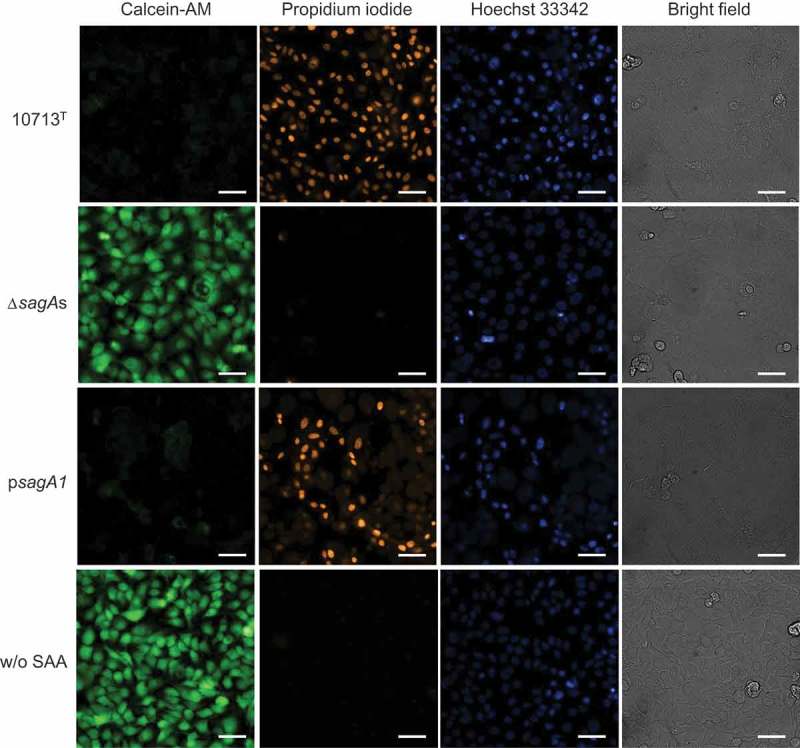
The secreted SLS from β-hemolytic *S. anginosus* subsp. *anginosus* expressed cytotoxicity against HSC-2. HSC-2 was cultured in the bottom of Transwell and each tested strain was cultured in the upper layer of Transwell for 18 h at 37°C under 5% CO_2_ . After co-cultivation, the cells were stained with calcein-AM, propidium iodide, and Hoechst 33342, and then observed using IN Cell Analyser 6000. 10713^T^, β-hemolytic *S. anginosus* subsp. *anginosus* NCTC10713^T^; Δ*sagA*s, *sagA1* and *sagA2* double deletion strain of NCTC10713^T^; p*sagA1, sagA1*-gene *trans*-complemented strain of Δ*sagA*s; w/o SAA, HSC-2 without co-cultivation. The scale bar shows 30 μm.

In order to confirm whether the cytotoxicity against HSC-2 was due to the “secreted” SLS from β-SAA, the Transwell assay was conducted. Almost all of the cells showed positive staining with propidium iodide (PI) and almost negative staining with calcein-AM after co-cultivation with the β-hemolytic strain, either NCTC10713^T^ or *sagA1*-complemented strain of Δ*sagA*s, p*sagA1* (Figure 5). However, a contrasting result was observed after the co-cultivation with the Δ*sagA*s strain, that is, a negative staining with PI and a positive staining with calcein-AM (Figure 5). These results showed that the cytotoxicity of HSC-2 observed in the co-cultivation with the β-hemolytic strains was evidently caused by the secreted SLS.

### Time-course investigation of the SLS-dependent membrane disruption in HSC-2

**Figure 6.**
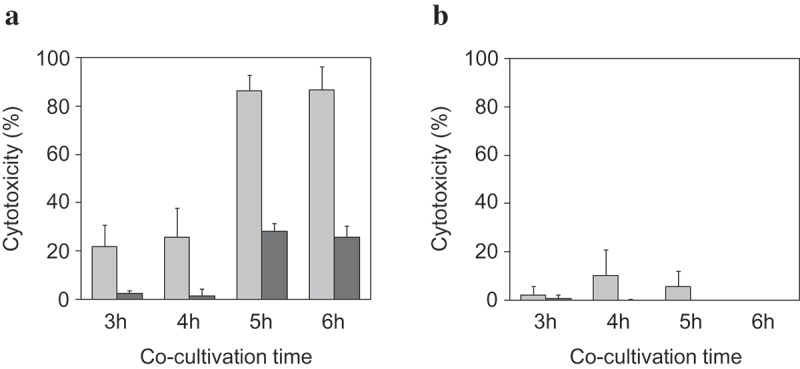
The time-dependent cytotoxicity of the SLS from β-hemolytic SAA against HSC-2. HSC-2 was co-cultivated with β-hemolytic *S. anginosus* subsp. *anginosus* NCTC10713^T^**(a)** or the non-hemolytic *sagA1* and *sagA2* double deletion strain of NCTC10713^T^ (Δ*sagA*s) **(b)**. The time-dependent cytotoxicity was evaluated using the CellTox^TM^ green cytotoxicity assay (light-gray bar) and CytoTox-ONE^TM^ homogeneous membrane integrity assay (dark-gray bar) from 3 h to 6 h co-cultivation. The results are shown as the percentage of cytotoxicity with standard deviation (SD) to 0% viable control (n = 3).

The time-course observation of SLS-dependent cytotoxicity of β-SAA was further investigated with a focus on the disruption to the cytoplasmic membrane. For this purpose, two kinds of evaluation systems were adopted: one is CytoTox-ONE Homogeneous Membrane Integrity Assay to assess the release of the lactate dehydrogenase (LDH), and the other is CellTox Green Cytotoxicity Assay to assess the internalization of the small chemical compound by the disruption to the cytoplasmic membrane. Consequently, time-dependent disruption to the cytoplasmic membrane was observed by co-cultivation with the SLS-producing strain NCTC10713^T^ (Figure 6(a)). The cytotoxicity evaluated by the CellTox Green Cytotoxicity Assay was observed at a relatively early stage of investigation, and obvious cytotoxicity (90% or more cell death) was observed after 5 h or longer co-cultivation (Figure 6(a), light-gray bar). Contrary to this, the faint-level of cytotoxicity observed until 4 h co-cultivation with NCTC10713^T^ and lower cytotoxicity began to be observed after 5 h or longer co-cultivation as evaluated by the CytoTox-ONE Homogeneous Membrane Integrity Assay (Figure 6(a), dark-gray bar). No obvious cytotoxicity was observed after the co-cultivation with the non-hemolytic Δ*sagA*s strain even when evaluated in both assay systems (Figure 6(b)). The difference in the detectability between the two assay systems is due to the detection principle for each assay system: CellTox Green Cytotoxicity Assay is based on the detection of the disruption to the cytoplasmic membrane with high sensitivity by the infiltration of small molecules across the disrupted membrane. With the LDH release-based CytoTox-ONE Homogeneous Membrane Integrity Assay it is difficult to detect such SLS-dependent disruption to the cytoplasmic membrane without large-scale pore or breakdown.

## Discussion

We have been investigating the pathogenic potential of β-hemolytic AGS, and had revealed that SLS is the sole hemolytic factor of the β-hemolytic AGS, except in *S. intermedius* that secretes the protein hemolysin intermedilysin [10,12]. In addition, we had revealed that the β-hemolytic *S. constellatus* subsp. *constellatus* strain W277 showed the SLS-dependent cytotoxicity in human hepatocellular carcinoma cell HepG2 [12]. However, another group reported that the SLS produced from SAA could not induce the cytotoxicity in the human acute monocytic leukemia cell line THP-1 and human granulocytes [26]. According to another report on the function of LLS, a homolog of SLS secreted from *L. monocytogenes*, LLS does not contribute to tissue injury and virulence in the host organs of *L. monocytogenes* [29]. Thus, in order to clarify the virulence potential of the ambiguous SLS, particularly from β-hemolytic *S. anginosus* subsp. *anginosus* (β-SAA), we tried to reveal the nature of SLS from β-SAA by evaluating its contribution to the cytotoxicity against human cell lines.

In this study, the bacterial cell number-dependent cytotoxicity in human oral squamous cell carcinoma cell line HSC-2 was observed under the co-cultivation with β-SAA strains (Figure 1(g)). From the microscopic observation, some blebs and the obvious morphological changes, probably due to the leakage of cellular contents by the secreted SLS, were observed by co-cultivation with β-SAA (Figure 1(a-d)). Although weak cytotoxicity was detected by the co-cultivation with a higher amount of the non-hemolytic SAA strain NCTC11169 (Figure 1(g)), no bleb was observed (Figure 1(e)). It appears that the weak cytotoxicity of non-hemolytic NCTC11169 may be due to some products such as the metabolites produced during the bacterial growth. Such SLS-dependent cytotoxicity was also observed with the genetically modified strains, Δ*sagA*s and p*sagA1* (Figure 2, Figure S1). Moreover, the investigation of the complementation of the Δ*sagA*s strain should be done using the strain complemented with both the *sagA1* and *sagA2* genes. However, the strain complemented with both *sagA1* and *sagA2* showed slightly impaired growth and hemolytic activity, compared to the parent strain NCTC10713^T^ and other genetically modified strains (Figure S3). Due to these observations, we adopted the *sagA1*-gene complemented strain p*sagA1* for the subsequent assays. Because even the p*sagA1* strain that produces only SagA1 (one of the SLS precursors encoded by the *sagA1* gene) showed the obvious cytotoxicity in HSC-2 (Figure 2(b)), it suggested that NCTC10713^T^ causes SLS-dependent cytotoxicity by the mature product derived from SagA1 under the co-cultivation condition. The reason for the growth deficiency observed in the *sagA1* and *sagA2* gene-complemented strain is under investigation.

Though the culture supernatant of β-SAA could not induce the acute cytotoxicity in HSC-2, a sub-acute effect was observed (Figure 4, Figure S2). Hence, the difference between the cytotoxicity against erythrocytes and culture cells may be due to the membrane repair mechanism equipped in the nucleated culture cells, which is absent in the erythrocytes. In addition, according to the Transwell assay (Figure 5), it was confirmed that the SLS secreted from β-SAA acted on the culture cells and induced the cytotoxicity in an SLS-dependent manner. These results showed that the SLS secreted from β-SAA functions as a cytotoxic factor at least *in vitro*. However, a recent report described that no difference could be observed between the hemolytic wild-type SAA and the non-hemolytic *sagB* mutant, at any MOI or time point by the LDH leakage assay for THP-1 and human granulocytes; and hence, concluded that the *S. anginosus* SLS is a broad-range hemolysin without cytolytic activity [26]. The difference in the experimental design between this previous report and ours were as follows: Firstly, the composition of the co-cultivation medium is different. In our study, 10% (v/v) of BHI broth was added to support the growth of the tested strain in the host cell culture conditions. In the condition using this co-cultivation medium, SLS is effectively produced in a growth-dependent manner for the tested strains (Figure S3). Hence, this condition is suitable for monitoring of SLS-dependent cytotoxicity to the cells *in vitro*. In addition, it is thought that some cellular contents leaked from the damaged cells help the growth of the tested strains; thus, the production of SLS is accelerated and the cytotoxicity to the cells is enhanced. Secondly, the method for the cytotoxicity assay is also different. The previous report evaluated the cytotoxicity using the LDH leakage method [26]. By this method, the cytotoxicity is detected at a relatively later stage of the cytoplasmic membrane disruption, which enables the leak of the cytoplasmic protein LDH (about 35 kDa of monomer, and the functional tetramer is about 140 kDa). However, it is difficult to assume that the critical cytoplasmic membrane disruption enabled to leak LDH which is induced at the early stage of co-cultivation with β-SAA, because of the cytotoxic character of SLS thought not to be rapid and drastic as shown in hemolysis by SLS. This idea is also supported by our results that the secreted SLS did not show an acute but a sub-acute effect (Figure 4). We adopted the CellTox-Green assay system for the cytotoxicity assay. This system enables to investigate the cytoplasmic membrane disruption by the infiltration of small molecules into cells through the damaged membrane. Using this system, the SLS-dependent cytotoxicity was successfully detected at a relatively early stage of co-cultivation with β-SAA, and an obvious time-lag in the detection of cytotoxicity was observed (Figure 6). Because the SLS-dependent cytotoxicity of β-SAA was also observed on THP-1 using the CellTox Green assay system (Figure 3), it was shown that the cytotoxicity of β-SAA by SLS is not limited to HSC-2. Based on these results, it is suggested that the SLS-dependent cytotoxicity can be established *in vivo* in the conditions that are suitable for the growth of β-SAA and the SLS secretion at their infected sites.

In *S. pyogenes*, it was reported that the *cre* sequence existed in the promoter region of the *sag* operon and it was suggested that the carbon catabolite repression contributed to the process of SLS production [30]. Recently, it has been reported that the SLS production from β-SAA is also regulated by the catabolite control protein CcpA [31]. These results suggest that the conditions for SLS production will be strongly affected by the environment in their habitats or infected sites. In addition, because our result showed that the SLS-dependent cytotoxicity of β-SAA is not high and is caused by a continuous action of secreted SLS (Figure 4), it is suggested that the cytotoxicity of β-SAA could be established by the continuous production of SLS at the infection sites of β-SAA. The AGS species including SAA is thought to be one of the opportunistic pathogens against humans and often escape from the human normal immune system to cause infection *in vivo*. Therefore, SLS may be a risk factor for infection caused not only by pyogenic streptococci such as *S. pyogenes* but also by the SLS-producing β-hemolytic sub-group of AGS. This prediction also suggests that suppression of SLS production or inactivation of SLS would help us to prevent the infective diseases caused by the SLS-producing AGS strains.

Our present investigation on the cytotoxicity of SLS produced from β-SAA was conducted *in vitro*. However, to reveal the mechanism of SLS-dependent cytotoxicity and virulence of β-SAA, further investigations *in vivo* would be required. In fact, it was reported that the contribution of SLS to the bacterial survival, neutrophil infiltration, and diffuse tissue necrosis is observed for the β-hemolytic group G streptococcus [32], and the contribution of SLS and streptolysin O to the early stages of infection and induction of necrotic lesions in mice is also observed for *S. pyogenes* [33]. In addition, it is also reported that the virulence factor of *S. pyogenes* is detected in some strains of *S. anginosus* [2]. Based on these accumulated recent findings on SLS and AGS, the underestimation in virulence of AGS, including SAA, as an opportunistic pathogen may increase the risk of misdiagnosis, prevention, and treatment of the infectious diseases caused by such a pathogenic sub-group of AGS producing the virulence factor(s) including the cytotoxic factor(s) such as SLS. In order to reveal and recognize the original pathogenicity of the β-hemolytic sub-group of AGS, further investigation is currently in progress.

## Data Availability

The authors confirm that the data supporting the findings of this study are available within the article and its supplemental materials.
