# Supplementary material for: β-Hemolytic Streptococcus anginosus subsp. anginosus causes streptolysin S-dependent cytotoxicity to human cell culture lines in vitro
Source: J Oral Microbiol. 2019 May 8;11(1):1609839. doi: 10.1080/20002297.2019.1609839 (PMC6508071; doi:10.1080/20002297.2019.1609839)
Supplement: Supplemental Material [file ZJOM_A_1609839_SM2264.pdf]

Supplemental Information

β-Hemolytic *Streptococcus anginosus* subsp. *anginosus* causes streptolysin S-dependent cytotoxicity to human culture cell lines *in vitro*

This file includes:

Supplemental Materials and Methods

Figure S1

Figure S2

Figure S3

Supplemental References

Supplemental Materials and Methods

Construction of trans-complemented strains

The vectors for *trans*-complementation were constructed using pMX2 [1]. Briefly, the region from the promoter of *sag* operon to *sagA2* (for both *sagA1* and *sagA2* complementation) or the region from the promoter of *sag* operon to *sagA1* (for *sagA1* complementation) was digested with *Eco*RI and *Pst*I from the vectors previously constructed [2], and inserted into the pMX2 digested with the same restriction enzymes. Each vector was introduced into the competent cells of *Escherichia coli* MC1061 by heat treatment (42 °C, 30 s), then selected on Luria-Bertani (LB) plate containing 20 μg/mL chloramphenicol. After confirmation of the insertion of the desired fragment by restriction enzyme digestion and PCR, the vector was purified and introduced into the mutant strain of *S. anginosus* subsp. *anginosus* without both the *sagA1* and *sagA2* genes (Δ*sagA*s), in the presence of competence-stimulating peptide (CSP) as described previously [2].

Growth dependent hemolytic property

The time-course investigation of the growth and hemolytic activity of the tested strains was carried out according to the method previously described [3]. Briefly, the tested strains were pre-cultured in BHI broth (for NCTC10713^T) or BHI broth containing 4 μg/mL of chloramphenicol (for *trans*-complemented strains). Subsequently, each bacterial culture was adjusted to the optical density at 600 nm (OD_{600}) of 0.01 with co-cultivation medium [EMEM (FUJIFILM Wako, Osaka, Japan) containing 10% (v/v) of inactivated fetal bovine serum (FBS), 10% (v/v) of BHI, and 50 mM HEPES (pH7.4)] and incubated at 37 °C under 5% CO_2 atmosphere. Both the growth (OD_{600}) and the hemolytic activity of the culture were measured every 2 h. For the measurement of hemolytic activity, the bacterial culture collected at each time point was centrifuged (15,400 × g, 5 min) and the supernatant was used for the assay. The sterile horse blood was purchased from Nippon Bio-Supp. Center (Bunkyo-ku, Tokyo, Japan) and the PBS-washed erythrocytes were added to the assay mixtures at the final concentration of 0.5% (v/v). To obtain the complete hemolysis control, the PBS-washed erythrocytes were suspended in the sterile pure water. And the sample of PBS-washed erythrocytes suspended in the sterile PBS was used as the non-hemolysis control. After incubation at 37 °C for 1 h, each sample was centrifuged (800 × g, 5 min) and dispensed as 0.2 mL into the well of 96-well plate. The absorbance at 540 nm was measured using microplate reader (Multiskan™ FC, Thermo Scientific, Waltham, MA, USA). The hemolytic activity was calculated as described previously [4].

Figure S1.

a b c

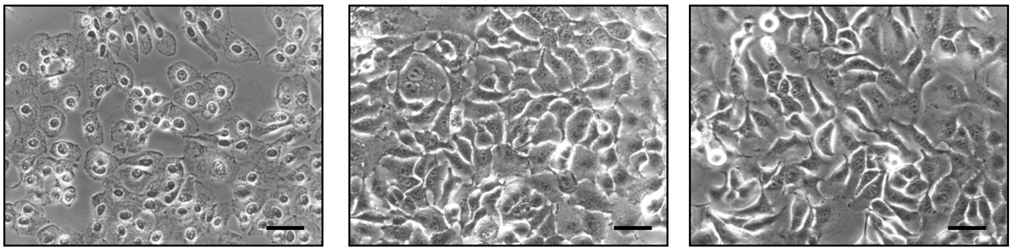

Figure S1. The morphological observation of HSC-2 after co-cultivation with *S. anginosus* subsp. *anginosus* NCTC10713^T and its non-hemolytic isogenic mutant strain of Δ*sagA*s strain. HSC-2 was co-cultivated with higher cell-density [OD_{600} = 0.1; multiplicity of infection (MOI) is about 1600 in this condition] of the *S. anginosus* subsp. *anginosus* NCTC10713^T **(a)** and Δ*sagA*s strain (*sagA1* and *sagA2* double-deletion mutant of *S. anginosus* subsp. *anginosus* NCTC10713^T) **(b)** for 4 h according to the description in the figure caption for Fig. 1. The morphology of the HSC-2 without co-cultivation of bacteria was also observed **(c)**. The morphological observation was carried out using the inverted microscope IX71 (Olympus) equipped with DP72 (Olympus) after removal of the bacterial cells. The scale bar shows 50 μm.

Figure S2.

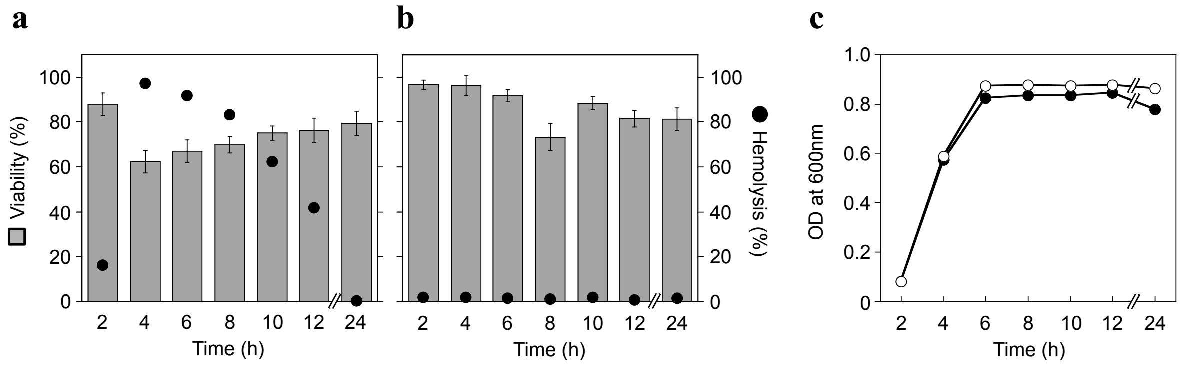

Figure S2. The viability of HSC-2 incubated with the culture supernatant of the tested strains obtained from the different time point in culture. HSC-2 cells were incubated with the culture supernatant of the tested strains [*S. anginosus* subsp. *anginosus* NCTC10713^{T} **(a)** and its Δ*sagA*s strain (*sagA1* and *sagA2* double-deletion mutant of *S. anginosus* subsp. *anginosus* NCTC10713^{T}) **(b)**] obtained from the different time point shown under the horizontal axes and incubated for 24 h. Subsequently, the viability was evaluated using CCK-8. The results are shown as the percentage of viability with standard deviation (SD) (n = 3) to 100% of the viable control of the HSC-2 with no treatment with bacterial culture supernatant. The hemolytic activity of each culture supernatant against horse erythrocytes was also measured as described in the "Supplemental Materials and Methods". The results are shown as the percentage with standard deviation (SD) (n = 3) with black circles in the graphs **(a)** and **(b)**. In addition, the growth of the tested strains (black circle, *S. anginosus* subsp. *anginosus* NCTC10713^{T}; white circle, Δ*sagA*s strain) in the co-cultivation medium at 37 °C under 5% CO_2 atmosphere was monitored by the OD at 600 nm **(c)**. These experiments were done twice and the representative results are shown.

Figure S3.

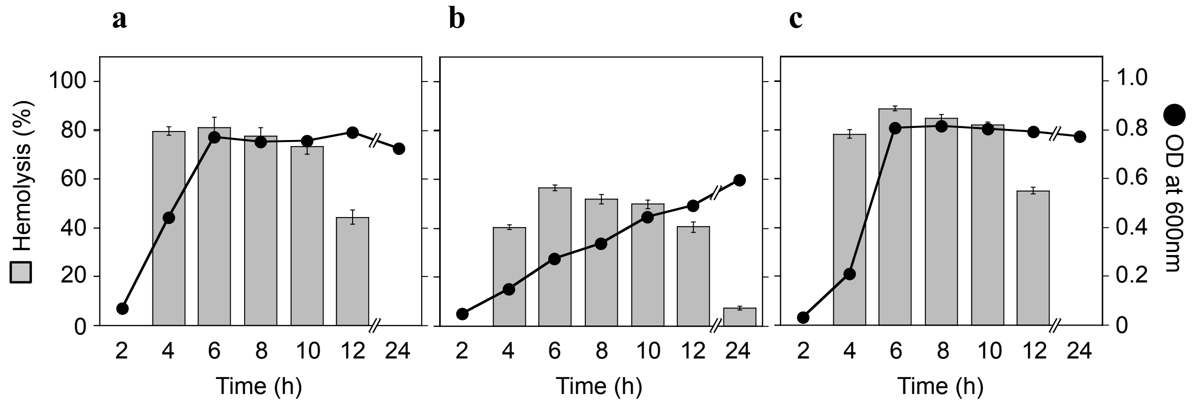

Figure S3. Time-course investigation for the growth and the hemolytic activity of the tested strains. *S. anginosus* subsp. *anginosus* NCTC10713^{T} **(a)** and the gene-complemented strains *in trans* [**(b)**, *sagA1* and *sagA2* complemented strain; **(c)**, *sagA1* complemented strain] were grown in the co-cultivation medium at 37 °C under 5% CO_2 atmosphere. The sampling was carried out every 2 h and the OD at 600 nm was measured (line graph with black circle). The hemolytic activity of the culture supernatant (gray bar) was also measured as described in the "Supplemental Materials and Methods" and the results were shown as the percentage with standard deviation (SD) (n = 3). This experiment was done twice and the representative results are shown.

Supplemental References

[1] Takamatsu D, Arai R, Miyoshi-Akiyama T, Okumura K, Okura M, Kirikae T, Kojima A, Osaki M. Identification of mutations involved in the requirement of potassium for growth of typical *Melissococcus plutonius* strains. Appl Environ Microbiol. 2013; 79:3882-3886.

[2] Tabata A, Nakano K, Ohkura K, Tomoyasu T, Kikuchi K, Whiley RA, Nagamune H. Novel twin streptolysin S-like peptides encoded in the *sag* operon homologue of beta-hemolytic *Streptococcus anginosus*. J Bacteriol. 2013; 195:1090-1099.

[3] Tabata A, Sato Y, Maya K, Nakano K, Kikuchi K, Whiley RA, Ohkura K, Tomoyasu T, Nagamune H. A streptolysin S homologue is essential for β-haemolytic *Streptococcus constellatus* subsp. *constellatus* cytotoxicity. Microbiology. 2014; 160:980-991.

[4] Nagamune H, Ohnishi C, Katsuura A, Fushitani K, Whiley RA, Tsuji A, Matsuda Y. Intermedilysin, a novel cytotoxin specific for human cells secreted by *Streptococcus intermedius* UNS46 isolated from a human liver abscess. Infect Immun. 1996; 64:3093-3100.
